# The role of remote ischemic preconditioning on postoperative kidney injury in patients undergoing cardiac and vascular interventions: a meta-analysis

**DOI:** 10.1186/1749-8090-8-43

**Published:** 2013-03-09

**Authors:** Lan Li, Guogang Li, Chaohui Yu, Youming Li

**Affiliations:** 1 Department of Gastroenterology, The First Affiliated Hospital, College of Medicine, Zhejiang University, Hangzhou 310003, People’s Republic of China; 2 Department of Surgery, The Second Affiliated Hospital, College of Medicine, Zhejiang University, Hangzhou 310009, People’s Republic of China

**Keywords:** Remote ischemic preconditioning, Cardiac and vascular interventions, Acute kidney injury, Meta-analysis

## Abstract

The objective of this study was to perform a meta-analysis of randomized controlled trials (RCTs) investigating whether a remote ischemic preconditioning (RIPC) protocol provides renal protection to patients undergoing cardiac and vascular interventions. Searches were conducted in the databases PUBMED, EMBASE and Cochrane Library. RCTs that fulfilled the inclusion criteria and addressed the clinical questions of this analysis were further assessed. We identified ten studies with a total of 924 patients undergoing cardiac and vascular interventions with or without RIPC. There was a significantly lower incidence of acute kidney injury in the RIPC group compared with control group using the fixed effect model (RR 0.69, 95% CI 0.53 to 0.90, *P* = 0.007), but not with the random effects model (RR 0.73, 95% CI 0.50 to 1.06, *P* = 0.10). There was no difference in the levels of renal biomarkers, incidence of renal replacement therapy, mortality, hospital stay, and intensive care unit stay between two groups. In conclusion, there is no enough evidence that RIPC provided renal protection in patients undergoing cardiac and vascular interventions. Large-scale RCTs are necessary to confirm the potential role of RIPC on renal impairment.

## Background

Acute kidney injury (AKI) affects up to 45% of patients undergoing cardiac surgery, percutaneous coronary intervention, and vascular surgery, and requires postoperative renal replacement therapy in nearly 1 to 2% [1,2]. Patients who develop AKI following cardiac and vascular interventions continue to have increased morbidity and mortality and prolonged stays in intensive care unit (ICU) and hospital [3,4].

Several different injury pathways including exogenous and endogenous toxins, metabolic and neurohormonal factors, renal ischemia and inflammatory surgical response contribute to the development of AKI during cardiac and vascular interventions [5-7].

Numerous clinical trials of pharmacologic interventions have been used to prevent AKI in patients following cardiac and vascular surgery; however, these studies have also been a disappointment [8-10].

Remote ischemic preconditioning (RIPC) is a phenomenon in which a brief ischemia and reperfusion in distant tissues protects a critical target organ or tissue from a subsequent episode of lethal ischemia and reperfusion through either neuronal or humoral pathway [11-13]. Although the kidneys are not directly exposed to ischemia-reperfusion injury, RIPC might preserve kidney function in patients undergoing cardiac and vascular interventions through blocking free radical production and attenuating the inflammatory response involved in pathogenesis of AKI [6,7,14]. This technique of RIPC has significant potential to decrease ischemic injury of other organs in patients undergoing cardiac and vascular interventions.

The studies regarding the protective effect of RIPC against AKI in patients undergoing cardiac and vascular interventions were limited, and the results remained controversial and contradictory. Therefore, we performed a meta-analysis to investigate whether a RIPC protocol provides renal protection to patients undergoing cardiac and vascular interventions.

## Methods

### Study design

Studies were accepted based on the following criteria: study design – randomized controlled trial (RCT); study population – adults who underwent cardiac and vascular interventions; intervention – RIPC (irrespective of the duration, timing, and the vessel occluded to provide the ischemic preconditioning stimulus); comparison intervention – usual treatment without RIPC; primary outcomes – development of AKI, initiation of renal replacement therapy, renal biomarkers; secondary outcomes – mortality, hospital stay, ICU stay. We excluded review articles, retrospective analyses, case reports as well as studies that were only reported as abstracts. If any of data were insufficient or missing, we contacted the authors to obtain information about missing data. This study was carried out in strict accordance with the Helsinki Declaration. The protocol was approved by the medical ethics committee of the first affiliated hospital of Zhejiang University (Permit Number: 2012-183).

### Literature search

Two investigators independently identified the published RCT from the PubMed (US National Library of Medicine, Bethesda, MD, USA) (1980 to present), EMBASE (Reed Elsevier PLC, Amsterdam, The Netherlands) (1980 to present) and Cochrane Library databases. In addition, we scanned the bibliographies of all relevant studies and recent review articles for further potential references. We also searched for unpublished and ongoing trials in clinicaltrials.gov and controlled-trials.com. The search terms were “ischemic preconditioning” (subject heading); “cardiovascular surgical procedures” (subject heading); “randomized controlled trial” (publication type); “controlled clinical trial” (publication type); “remote ischemic preconditioning” (text keywords) and “randomized controlled trial” (text keywords). No language restrictions were applied to any search strategies. There was complete consensus among two investigators on the final results.

### Data extraction

Data extraction was performed by two independent observers using standardized forms. Recorded data included the demographic characteristics of the patients, procedures of operation, protocol for RIPC, the incidence of AKI, the incidence of hemodialysis or hemofiltration, mortality, ICU and hospital stays, serum creatinine levels before and after surgery, glomerular filtration rate (GFR) before and after surgery. The studies included in the meta-analysis were assessed for methodological quality using the Jadad composite scale. According to this scale, a low-quality study should score 2 or less points and high-quality study should score 3 or more points [15]. Allocation concealment was assessed with the classification of the Cochrane Collaboration. Disagreements were resolved by contacting authors or reaching a consensus.

### Statistical analysis

If several trials were available for a specific topic, meta-analysis was conducted using the software RevMan 5.1 (provided by the Cochrane Collaboration, Oxford, UK). We calculated the risk ratio (RR) with 95% confidence interval for dichotomous outcomes and mean difference (MD) with 95% confidence interval for continuous outcomes. We assessed statistical heterogeneity using the *χ*^2^ tests and determined the percentage of total variation across studies using the Higgins I^2^ statistic. The fixed-effect model and the random-effects model were used to pool studies. In case of discrepancy between the two models we reported both results; otherwise we reported only the results from the fixed effect model.

## Results

### Literature search

**Figure 1:**
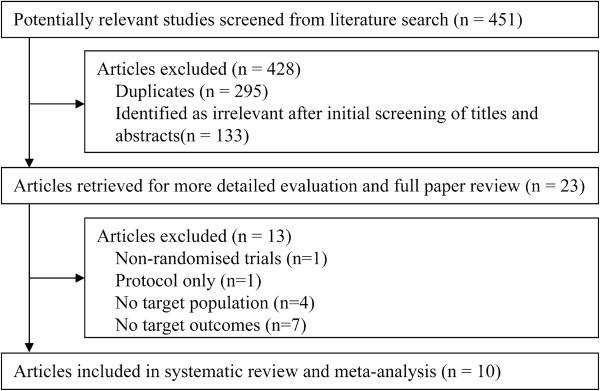
Identification of trials for inclusion.

The search strategy generated 451 studies. After exclusion of 295 duplicates and 133 clearly irrelevant papers through reading abstracts, 23 papers were retrieved for further assessment. Of the 23 references, 13 were excluded because of retrospective analysis of 2 prior studies (n = 1), protocol only (n = 1), no target population (n = 4), no target outcomes (n = 7). Finally, a total of ten papers were eligible for this meta-analysis [16-25] (Figure 1).

### Study characteristics

A total of 924 participants were enrolled in the ten studies, including six studies in patients undergoing cardiac surgery, one studies in percutaneous coronary intervention, and three studies in vascular surgery. 464 patients were randomised to the RIPC group, and 460 to the control group. There were no significant differences between two groups with regard to age, sex and preoperative renal status. The important characteristics of the included studies are shown in Table 1.

**Table 1:** **Demographic data of studies included in meta**-**analysis** (**RIPC group**/**control group**)

	**No. of patients**	**Mean age (years)**	**Males (%)**	**Comorbidities**		**Baseline creatinine (μmol/L)**	**Surgical procedures**	**RIPC methods**
				**Hypertension, no.**	**DM, no.**			
Ali et al. [16]	41/41	74/75	93/93	21/26	2/2	102/101	Abdominal aortic aneurysm repair	Crossclamping of the iliac arteries
Hoole et al. [17]	104/98	63.2/61.8	81/76	53/51	24/20	–	PCI	An inflatable tourniquet around the limbs
Walsh et al. [18]	18/22	74/76	100/100	8/12	3/2	95/94	Endovascular aneurysm repair	An inflatable tourniquet around the limbs
Walsh et al. [19]	22/18	75/72	73/100	12/16	1/0	97/88	Abdominal aortic aneurysm repair	Crossclamping of the iliac arteries
Rahman et al. [20]	80/82	63/65	89/88	44/52	3/0	98.1/96.4	CABG	An inflatable tourniquet around the limbs
Choi et al. [21]	38/38	57/60	39/39	8/10	1/4	80.4/81.3	CABG, Valve surgery, Bentall procedure	An inflatable tourniquet around the limbs
Thielmann et al. [22]	27/26	63.4/64.1	85/85	25/24	0/0	–	CABG	An inflatable tourniquet around the limbs
Zimmerman et al. [23]	59/59	62/65	41/40	44/50	24/21	82.2/84.0	CABG, Valve surgery	An inflatable tourniquet around the limbs
Lucchinette et al. [24]	27/28	59/62	96/86	19/20	–	91.7/88.0	CABG	An inflatable tourniquet around the limbs
Young et al. [25]	48/48	65.5/64.4	60/65	–	–	102/95	CABG, Valve surgery	An inflatable tourniquet around the limbs

DM, diabetes mellitus; PCI, percutaneous coronary intervention; CABG, coronary artery bypass graft.

### Quality of methods

The quality of the included studies was assessed using the Jadad score and allocation concealment classification of the Cochrane Collaboration. Eight of ten trials had high methodological quality and a low risk of bias [16-21,24,25]. The generation of a randomization list was adequate in eight trials [16-21,24,25]. The allocation concealment was adequate in eight trials [16-20,23-25]. Double blinding was performed in two trials [20,25]. All trials had a clear explanation for withdrawals and dropouts in each group. Six trials reported no withdrawals after randomization [16,18,20,21,24,25]; in four trials [17,19,22,23], 11.62% (48/413) of patients were withdrawn after randomization. Only one patient was lost to follow-up [17] (Table 2).

**Table 2:** **Methodological quality of trials included in meta**-**analysis**

	**Year**	**Randomization method**	**Blind**	**Explanation for withdrawals/drop outs**	**Jadad score**	**Allocation concealment**	**Intention-to-treat analysis**
Ali et al. [16]	2007	Computer-generated random numbers	Single blind	Yes	3	Sealed envelopes	Yes
Hoole et al. [17]	2009	Computer-generated random numbers	Single blind	Yes	3	Sealed envelopes	No
Walsh et al. [18]	2009	Computer-generated random numbers	Unclear	Yes	3	Sealed envelopes	Yes
Walsh et al. [19]	2010	Computer-generated random numbers	Unclear	Yes	3	Sealed envelopes	Yes
Rahman et al. [20]	2010	Computer-generated random numbers	Double blind	Yes	5	Sealed envelopes	Yes
Choi et al. [21]	2010	Computer-generated randomization table	Unclear	Yes	3	Unclear	Yes
Thielmann et al. [22]	2010	Unclear	Single blind	Yes	2	Unclear	Yes
Zimmerman et al. [23]	2011	A block randomization generated by the study coordinator	Single blind	Yes	2	Sealed envelopes	Yes
Lucchinette et al. [24]	2012	Computer-generated random numbers	Unclear	Yes	3	Sealed envelopes	Yes
Young et al. [25]	2012	A block randomization using online randomization sequence generator	Double blind	Yes	5	Sealed envelopes	Yes

### Effects of interventions

#### Incidence of acute kidney injury

**Figure 2:**
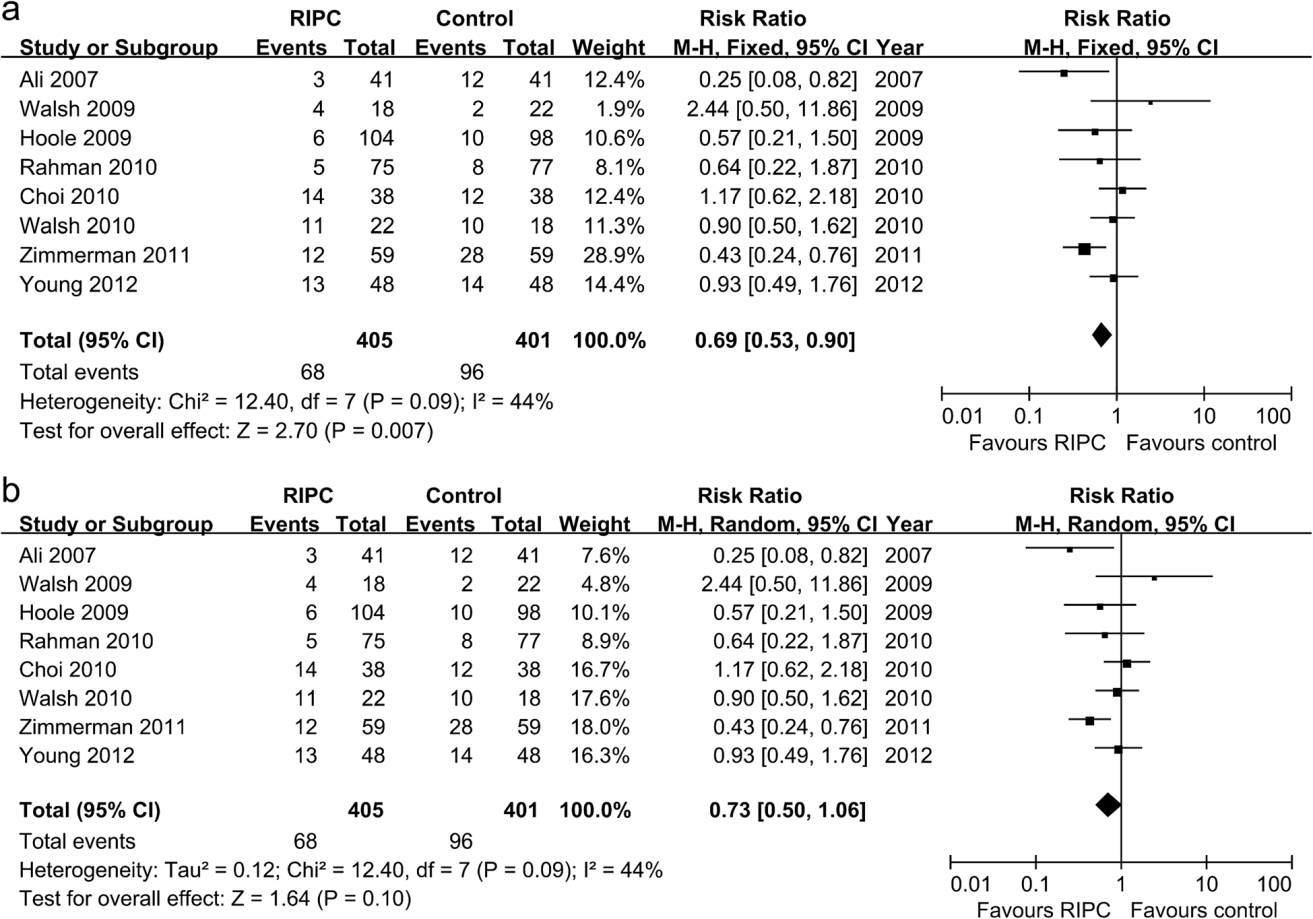
**Meta**-**analysis of relative risk for the incidence of AKI.** (**a**) Fixed effect model; (**b**) Random effect model.

Information on the incidence of AKI was available for eight trials included in the meta-analysis [16-21,23,25]. There was a significantly lower risk of AKI in the RIPC group compared with control group using the fixed effect model (RR 0.69, 95% CI 0.53 to 0.90, *P* = 0.007, Figure 2a), but not with the random effects model (RR 0.73, 95% CI 0.50 to 1.06, *P* = 0.10, Figure 2b). However, these findings should be regarded with caution since we used the definition of AKI applied by the investigators of the respective studies. The definitions included AKIN criterion [21,23], RIFLE criterion [25], peak serum creatinine level > 2 mg/dl [16], a postoperative decline in eGFR > 20% [18,19], and a postoperative rise in serum creatinine > 0.5 mg/dL [20]. There was a significant heterogeneity, with I^2^ of 44%. Futhermore we performed a subgroup analysis of the trials with AKIN criterion to evaluate the efficacy of RIPC for AKI. We observed that RIPC significantly reduced the risk of AKI with the fixed effect model (RR 0.65, 95% CI 0.43 to 0.98, *P* = 0.04), but not with the random effects model (RR 0.70, 95% CI 0.26 to 1.88, *P* = 0.48). Large-scale trials are necessary to investigate the potential effect of RIPC against AKI.

#### Biomarkers of kidney injury

**Figure 3:**
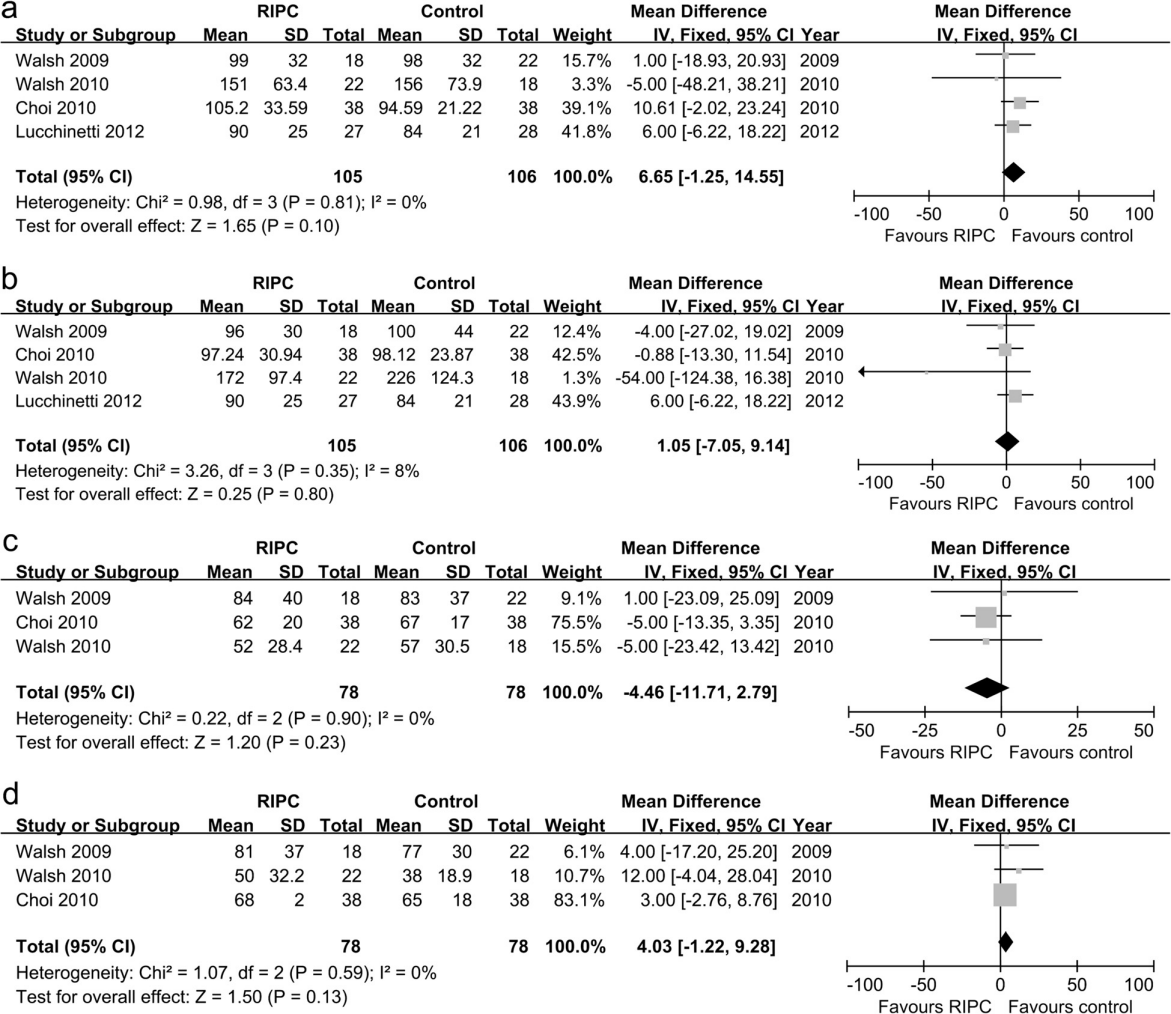
**Meta**-**analysis of mean difference for the levels of renal biomarkers.** (**a**) serum creatinine levels on the first postoperative day; (**b**) serum creatinine levels on the second postoperative day; (**c**) GFR values on the first postoperative day; (**d**) GFR values on the second postoperative day.

Available information on the data of serum creatinine for four trials was included in the meta-analysis [18,19,21,24]. The serum creatinine levels on the first postoperative day did not differ significantly between two groups (MD 6.65, 95% CI −1.25 to 14.55, *P* = 0.10, Figure 3a). Moreover, there was no significant difference in serum creatinine levels on the second postoperative day (MD 1.05, 95% CI −7.05 to 9.14, *P* = 0.80, Figure 3b).

Three trials collected the GFR values from the RIPC and control groups [18,19,21]. There was no difference in the GFR values between two groups on the first postoperative day (MD −4.46; 95% CI −11.71 to 2.79, *P* = 0.23, Figure 3c) and the second postoperative day (MD 4.03, 95% CI −1.22 to 9.28, *P* = 0.13, Figure 3d).

#### Renal replacement therapy

**Figure 4:**
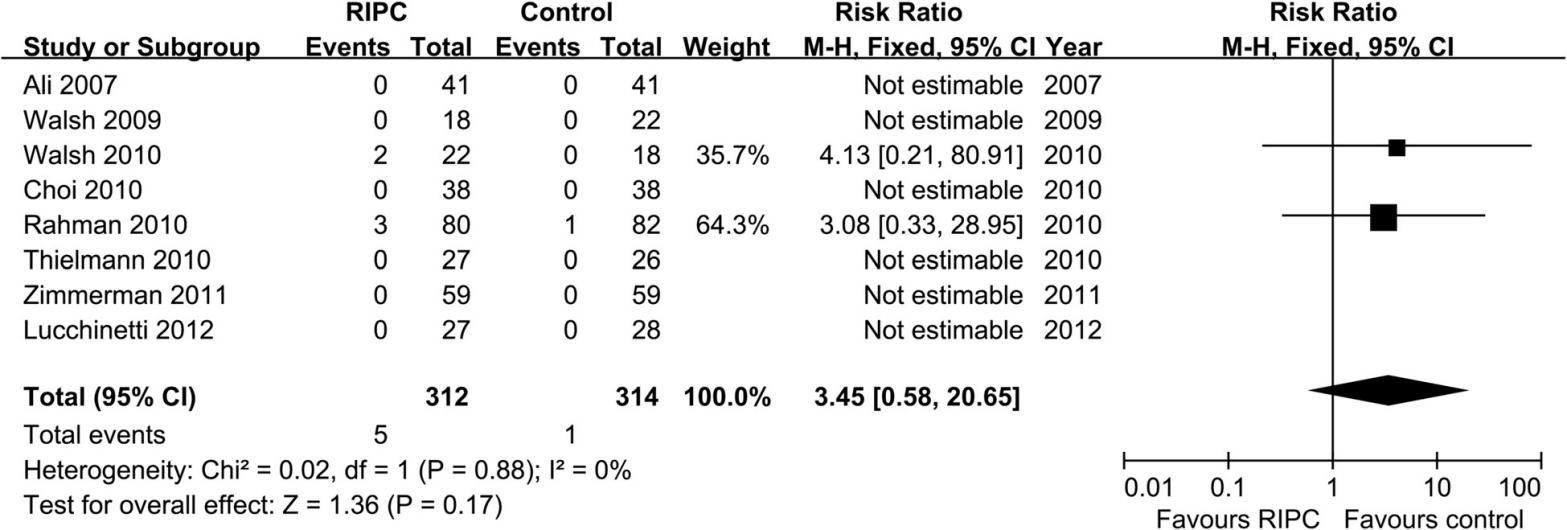
**Meta**-**analysis of relative risk for the incidence of renal replacement therapy.**

Eight trials evaluated renal replacement therapy as an outcome [16,18-24]. In six of the eight trials, none of patients required postoperative hemodialysis or hemofiltration. Two trials by Walsh et al. [19] and Rahman et al. [20] reported 6 patients who received renal replacement therapy (5 in the RIPC group and 1 in the control group). No significant difference was observed in the incidence of renal replacement therapy between two groups (RR 3.45, 95% CI 0.58 to 20.65, P = 0.17, Figure 4).

#### Mortality

**Figure 5:**
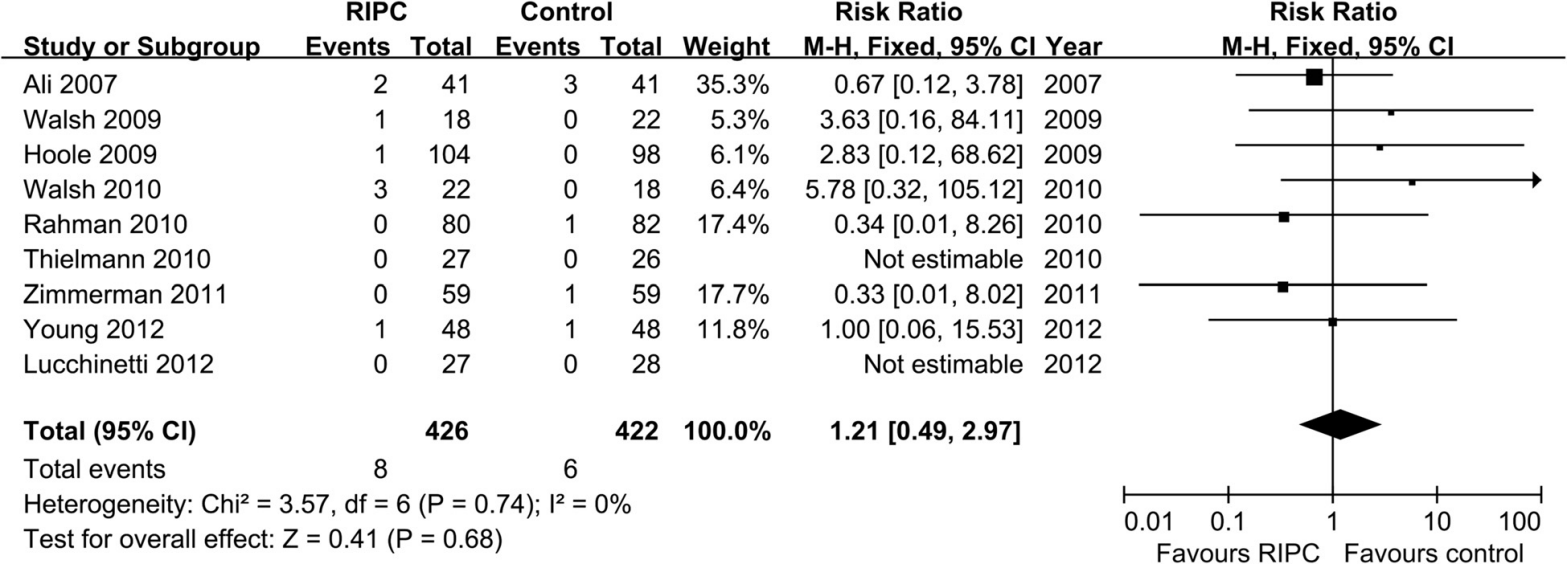
Meta-analysis of relative risk for mortality.

The mortality was reported in nine trials [16-20,22-25]. There was no statistically significant difference in the overall mortality between two groups (RR 1.10, 95% CI 0.49 to 2.97, *P* = 0.68, Figure 5).

#### Hospital stay

**Figure 6:**
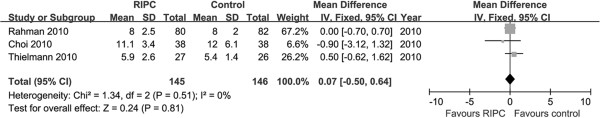
**Meta**-**analysis of mean difference for hospital stay.**

Available information on the hospital stay for three trials was included in the meta-analysis [20-22]. No statistically significant difference was observed in the hospital stay between two groups (MD 0.07, 95% CI −0.50 to 0.64, *P* = 0.81, Figure 6).

#### ICU stay

**Figure 7:**
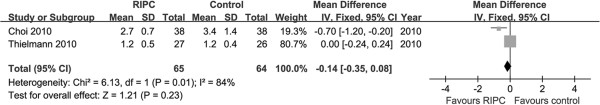
**Meta**-**analysis of mean difference for ICU stay.**

Information on the time of ICU stay for two trials was included in the meta-analysis [21,22]. There was no statistically significant difference in the ICU stay between two groups (MD −0.14, 95% CI −0.35 to 0.08, *P* = 0.23, Figure 7).

### Subgroup analysis

We performed the subgroup analysis for the patients undergoing cardiac surgery [17,20-25]. The RR for the incidence of AKI, hemodialysis requirement and mortality remained statistically stable. Moreover, a subgroup analysis of trials using tourniquet cuff around the limb for RIPC did not reveal any change in the incidence of AKI, hemodialysis requirement and mortality [17,18,20-25]. We did not perform a subgroup analysis of outcomes for renal biomarkers, hospital stay and ICU stay as few studies in the subgroup reported these outcomes.

### Sensitivity analysis

We performed a meta-analysis of high quality trials [16-21,24,25] to evaluate the efficacy of RIPC for renal protection. There was no significant difference between two groups for incidence of AKI, renal biomarkers, hemodialysis requirement, or mortality after excluding the trials with low quality.

## Discussion

RIPC prior to cardiac and vascular interventions has primarily been used to ameliorate heart ischemia-reperfusion injury [26]. The pooled results of these trials indicate a significant benefit of RIPC for decreasing the levels of myocardial necrosis markers and the incidence of perioperative myocardial infarction [27-31]. To date, whether RIPC can protect kidney function in patients undergoing cardiac and vascular interventions is still a controversial issue. The meta-analyses by D’Ascenzo et al. [29] and Brevoord et al. [30] which evaluated the effect of RIPC in the patients undergoing cardiac and vascular interventions concluded that serum creatinine levels were both not reduced by RIPC. Recently Desai et al. [32] published a meta-analysis including four RCTs of 115 patients undergoing vascular surgery, which showed no difference in the incidence of renal impairment between RIPC and controls. However, the meta-analysis by Alreja et al. [31] showed that RIPC significantly reduced the levels of serum creatinine in the first few days after cardiac and vascular interventions. These apparent inconsistencies may be due to limitations in few numbers of trials, small sample size and low to moderate methodological quality. Therefore, a further update of meta-analysis assessing the role of RIPC on renal protection in patients undergoing cardiac and vascular interventions should be performed. Compared with previous meta-analyses, our paper included the studies across a broader population over a longer time frame. Concerning renal end points, previous meta-analyses extracted either serum creatinine or incidence of AKI, whereas we tried to extract all renal outcomes from the included trials.

Our data demonstrate that there was a significantly lower incidence of AKI in the RIPC group by the fixed effect model, but no difference between two groups by the random effects model. The result remained unchanged in all subgroup analysis. In view of the size and quality of currently published trials, these data were not able to conclude whether RIPC has any beneficial or harmful effects on renal protection.

Furthermore, we assessed renal injury with postoperative measurement of serum creatinine. In contrast to our expectation, there was no difference in the serum creatinine levels between two groups. Although the most widely used biomarker for diagnosis of AKI is serum creatinine, serum creatinine is a late biomarker of AKI because it is not accurate in the acute care setting but rather in the steady-state which can take several days to reach. In addition, volume expansion during cardiopulmonary bypass in patients undergoing cardiac surgery may result in a fall of serum creatinine despite the presence of significant renal injury. Therefore, it is recommended that some potential early biomarkers for AKI also be examined in the future studies (e.g., interleukin-18, kidney injury molecule-1 and neutrophil gelatinase-associated lipocalin). We also assessed GFR values 24 and 48 hours after the surgery. Still no benefits were seen in patients treated with RIPC. Likewise, we did not find a significant reduction in hemodialysis requirement, mortality, hospital stay and ICU stay in the RIPC group compared with the control groups.

The studies included in this review had inconsistencies in the premedication, anesthesia, and surgical procedures. We intended to carry out subgroup analysis by type of interventions. Due to the limited number of trials included, only the group of studies in patients undergoing cardiac surgery was large enough to permit a subgroup analysis. We found that RIPC did not reduce the incidence of AKI in patients undergoing cardiac surgery. The hemodialysis requirement and mortality did not differ between the RIPC and control group. The present analysis does not support the hypothesis that RIPC provides kidney protection in patients undergoing cardiac surgery. We also made an effort to compare different protocols of RIPC. Although eight of ten studies used cuff inflation and deflation around the arm or leg for RIPC, crossclamping of the iliac arteries was used by Ali et al. [16] and Walsh et al. [19]; which RIPC protocol is truly the best for myocardial and renal protection is still uncertain. The results remained statistically stable in a subgroup analysis for the incidence of AKI, hemodialysis requirement and mortality after excluding trials using iliac artery clamping.

The meta-analysis suffers from the following limitations. First, there were only two double-blind trials included in our meta-analysis. In the other eight trials, only patients and laboratory investigators were blinded to randomization, whereas anesthetists and surgeons were aware of the participants’ group assignment. Therefore, the outcomes of this meta-analysis could be influenced by care providers’ awareness of study group assignment. Second, some of studies included only patients without diabetes mellitus, which is associated with a decreased risk of AKI. Therefore, the findings of this meta-analysis were not applicable to patients with diabetes mellitus because of the heterogeneity for study population. Third, as mentioned above, various definitions have been suggested for postoperative AKI. Although we have tried to account for this heterogeneity by using a random effects model and performing subgroup analysis, these data do not yet allow for definitive conclusions. Moreover, the definition of AKI used in these trials does not take into account the duration of serum creatinine elevation. These limitations may explain the heterogeneity between studies. Therefore, the result of the meta-analysis should be interpreted with caution and be regarded as hypothesis generating, rather than conclusion drawing.

## Conclusion

RIPC has no beneficial effect on the postoperative occurrence of AKI, renal biomarkers, hemodialysis requirement, mortality, or hospital and ICU stays during cardiovascular interventions. There is no evidence that RIPC provides renal protection in patients undergoing cardiac and vascular interventions. However, in view of the inherent limitations of meta-analysis extracted from currently published RCTs, our data should be regarded as exploratory with further studies needed in patients undergoing cardiac and vascular interventions. The future clinical studies should be designed to develop optimal RIPC procedures in accordance with operation type and elucidate the molecular mechanisms of RIPC.

## Abbreviation

AKI: Acute kidney injury; RCT: Randomized controlled trial; RIPC: Remote ischemic preconditioning; ICU: Intensive care unit; GFR: Glomerular filtration rate; RR: Risk ratio; MD: Mean difference; DM: Diabetes mellitus; PCI: Percutaneous coronary intervention; CABG: Coronary artery bypass graft.

## Competing interests

This work was supported by the Zhejiang Provincial Medical and Healthy Science Foundation of China [2009B043].

## Authors’ contributions

LL and YL participated in study design and RCTs assessment. GL and CY carried out data analysis. LL, GL, CY and YL participated in writing the manuscript. All authors read and approved the final manuscript.
